# Cigarette smoking is associated with an increase in blood monocytes in people with tuberculosis: A cross-sectional study

**DOI:** 10.1097/MD.0000000000030737

**Published:** 2022-09-16

**Authors:** Joseph Baruch Baluku, Martin Nabwana, Grace Kansiime, Edwin Nuwagira

**Affiliations:** a Division of Pulmonology, Kiruddu National Referral Hospital, Kampala, Uganda; b Makerere University Lung Institute, Kampala, Uganda; c Makerere University-John Hopkins University Research Collaboration, Kampala, Uganda; d Department of Internal Medicine, Mbarara University of Science and Technology, Mbarara, Uganda.

**Keywords:** cigarette, monocytes, neutrophils, smoking, TB, tuberculosis

## Abstract

The effect of smoking on immune responses in people with tuberculosis (TB) is not well elucidated. We aimed to compare peripheral blood counts of CD4+ and CD87 + T-lymphocytes, monocytes, and neutrophils and the CD4:CD8 ratio in TB patients with and without history of cigarette smoking. We further determined factors associated with current smoking. Participants with TB were consecutively enrolled in a cross-sectional study at a national TB treatment center in Uganda in 2018. We compared cell counts and the CD4:CD8 ratio using the median test among never smokers, past smokers (>6 months ago) and current smokers (≤6 months). Factors associated with current smoking were determined using logistic regression. A post hoc analysis for factors associated with an increase in the monocytes was also performed. Of 363 participants, there were 258 (71.1%) never smokers, 50 (13.8%) past smokers, and 55 (15.2%) current smokers. Most current smokers (49.1%) had a high sputum mycobacterial load. They also had the lowest body mass index and the highest axillary temperature. The median (interquartile range [IQR]) monocyte count among current smokers was 815 (540–1425) cells/mm^3^ and was significantly higher than that among past smokers (610 (350–900) cells/mm^3^, *P* = .017) and never smokers (560 [400–800] cells/mm^3^, *P* = .001). The monocyte counts positively correlated with the number of cigarettes smoked per day among current smokers (*R* = 0.43, *P* = .006). Current smokers also had higher neutrophil and CD4+ T-cell counts than never smokers. In a multivariable logistic regression model, an increase in the monocyte count was associated with current cigarette smoking (adjusted odds ratio [aOR] = 4.82, 95% confidence interval 1.61–14.39, *P* = .005). Similarly, current cigarette smoking was independently associated with an increase in the monocyte count (aOR = 1.80, 95% CI 1.39–2.32, *P* < .001). Cigarette smoking is associated with an increase in the blood monocytes in people with TB in a dose- and time-dependent manner. Further, current smoking is associated with an increase in neutrophils and CD4+ T-lymphocytes. The findings suggest that current smokers have systemic inflammation that is not necessarily beneficial to TB control in TB patients.

## 1. Introduction

In 2020, there were 10 million new tuberculosis (TB) cases and 1.3 million TB deaths globally.^[1]^ Cigarette smoking is an established risk factor for latent TB infection, active TB disease and TB-related mortality.^[2]^ It is estimated that smoking accounts for 18% of incident TB cases and 15% of TB mortality in TB high-burdened countries.^[3]^ Moreover, both current and past smoking are associated with higher odds of drug resistant TB.^[4]^ Recent meta-analyses report that smoking is associated with 51% higher likelihood of unfavorable TB treatment outcomes, including delayed culture conversion, and treatment loss-to-follow up.^[5,6]^

The rates of smoking are still high in TB burdened regions. In Africa, which accounts for a quarter of the global TB cases,^[1]^ the prevalence of smoking is up to 38% in the general population.^[7,8]^ A point estimate of the prevalence of smoking was reported to be 8.3% from demographic surveys of 25 African countries.^[9]^ Among people with active TB in Africa, smoking rates range from 26% in Uganda^[10]^ to 82.5% in South Africa.^[11]^ This highlights a high burden of smoking among people with TB for whom smoking cessation programs are not readily available.

Nicotine in tobacco attenuates innate immune responses against TB by decreasing expression of toll-like receptors and production of cytokines (interleukin [IL]—6 and 8 and tumor necrotic factor alpha [TNFα]) and chemokines by lung epithelial cells, macrophages and type 2 pneumocytes.^[12,13]^ Together, these mechanisms could explain why smoking increases the risk of TB infection and disease. In apparently healthy individuals, smoking also increases blood leucocytes, neutrophils, CD4 + T-lymphocytes, B-cells, and the CD4:CD8 ratio while concurrently reduces natural killer and CD8+ T cell counts, independent of genetic and environmental factors.^[14]^ Moreover, the elevation in lymphocytes, neutrophils, and monocytes is long term and is attributed to the chronic systemic inflammation induced by smoking.^[15]^ These observations are documented in the general population. The effect of smoking on blood immune cells among people with active tuberculosis is not well elucidated. Notwithstanding, baseline total leucocytes, lymphocytes, and neutrophils correlate with TB severity and predict TB treatment success.^[16,17]^ Further, neutrophilia in active TB is associated with 3-fold odds of mortality.^[18]^ The aim of this study was to compare peripheral blood counts of CD4+ and CD8+ T-lymphocytes, monocytes, and neutrophils and the CD4:CD8 ratio among TB patients with and without history of cigarette smoking. We further determined factors associated with current cigarette smoking.

## 2. Methods

The study was conducted in accordance with the Strengthening the Reporting of Observational Studies in Epidemiology (STROBE) guidelines.^[19]^

### 2.1. Study design, population, and setting

We analyzed data from a cross-sectional study conducted at the national tuberculosis treatment center, Mulago Hospital, in Uganda in 2018.^[20]^ Eligible participants were adults (≥18 years) who had pulmonary bacteriologically confirmed TB by either the Xpert MTB/Rif assay or auramine smear on sputum samples. Participants presenting at the facility were consecutively enrolled using the unit TB register as a sampling frame before initiating TB therapy. The national TB treatment center is in Kampala, the capital city of Uganda, and is a referral center for TB diagnosis and management. However, <30% are referral cases and majority of patients are primarily diagnosed from this health facility. The center manages both drug susceptible and resistant forms of TB at an outpatient and inpatient basis. Participants from the primary study were categorized based on self-reported history of cigarette smoking. As such, there were three unmatched categories of study participants: never smokers, past smokers, and current smokers. Past smokers were individuals who reported any history of cigarette smoking in >6 months before the study enrollment while current smokers reported smoking in ≤6 months. Self-reports of smoking correlate well with plasma levels of nicotine with sensitivity of 89% among people with TB.^[21]^

### 2.2. Study measurements and data collection

A detailed description of study measurements is provided elsewhere.^[20]^ Briefly, data were collected using a pretested questionnaire through a face-to-face interview. The questionnaire sought for demographic data, past medical history, previous TB history, and any history of alcohol use. Past and current smokers were asked to estimate the number of cigarettes smoked per day. Mycobacterial sputum load was determined by cycle threshold (Ct) values of the Xpert MTB/Rif assay as very low (Ct >28), low (Ct 22–28), medium (Ct 16–22), and high (Ct <16)^[22]^ and the sputum smear load as: very low (Scanty [1–9 acid fast bacilli [AFBs]/100 fields), low (1 + [1–9AFBs/10 fields]), medium (2 + [1–10AFBs/field]) and high (3+ [>10 AFBs/field]).^[23]^ Study participants underwent a brief physical examination for the axillary temperature, weight, and height. A study nurse drew 5 mL of venous blood using standard procedures. Blood samples were tested for HIV using immunochromatographic tests,^[24]^ full blood count using a haemoanalyser (Sysmex^®^ Automated hematology analyzer XN series—XN 1000), and CD4+ and CD8+ T-lymphocyte counts by flow cytometry using a flow cytometer (BD FACSCalibur™).

### 2.3. Statistical analysis

Data were entered in Epidata 3.1 and analyzed with STATA 16.0 (StataCorp, College Station, TX). Descriptive statistics were used to characterize the study population. Categorical variables were compared across the three groups (never smokers, past smokers, and current smokers) using Pearson chi-square or Fisher exact test. Continuous variables (weight, age, body mass index [BMI], and temperature) were compared across the three groups using one-way ANOVA. We compared blood neutrophil, monocytes, CD4+ and CD8+ T-lymphocyte counts and the CD4:CD8 ratio using the median test. Further, we used Pearson correlation coefficient to establish the correlation between the cell counts and CD4:CD8 ratio with the number of cigarettes smoked among past and current smokers. Logistic regression analysis was used to determine factors associated with current smoking. We constructed a multivariable logistic regression model for factors associated with current smoking by adding all variables with a *P* < .2 into the model and used backward stepwise regression to derive a parsimonious model. We intentionally kept the neutrophil, CD4+ and CD8+ T- lymphocyte counts, CD4:CD8 ratio and HIV status in the model as potential confounders. A post hoc analysis for factors associated with an increase in the monocyte count was also performed. A *P* < .05 was considered statistically significant for all analyses.

### 2.4. Ethics approval and informed consent

Participants provided written consent before enrollment in the study. The study was approved by the Makerere University School of Medicine Research and Ethics Committee (REC REF 2017-087).

## 3. Results

All 363 participants in the primary study were included in this analysis. Of these, there were 258 (71.1%) never smokers, 50 (13.8%) past smokers, and 55 (15.2%) current smokers.

### 3.1. Characteristics of study participants

Of all participants, 130 (35.8%) were co-infected with HIV. As shown in Table 1, current smokers were mostly male, had primary level education or less, high sputum mycobacterial load, chills, the highest median temperature, and the lowest body mass index (BMI).

**Table 1:** Characteristics of current, past, and never smokers with pulmonary TB.

Characteristic	Total N = 363 n (%)	Current smokers n = 55 n (%)	Past smokers n = 50 n (%)	Never smokers n = 258 n (%)	*P* value
Age, yr, mean (SD)	33 (11)	37 (12)	37 (13)	32 (10)	<.001
Sex					<.001
Male	223 (61.4)	51 (92.7)	42 (84.0)	130 (50.4)	
Female	140 (38.6)	4 (7.3)	8 (16.0)	128 (49.6)	
Education level					
≤Primary level	161 (44.4)	31 (56.4)	31 (62.0)	99 (38.4)	.001
≥Secondary level	202 (55.6)	24 (43.6)	19 (38.0)	159 (61.6)	
Alcohol use					
Ever used	187 (51.5)	44 (80.0)	44 (88.0)	99 (38.4)	<.001
Never used	176 (48.5)	11 (20.0)	6 (12.0)	159 (61.6)	
Type of residence					
Rural	118 (32.5)	14 (25.5)	13 (26.0)	91 (17.2)	.211
Urban	245 (67.5)	41 (74.5)	37 (74.0)	167 (64.7)	
HIV status					
Positive	130 (35.8)	13 (23.6)	21 (42.0)	96 (37.2)	.100
Negative	233 (64.2)	42 (76.4)	29 (58.0)	162 (62.8)	
Symptoms					
Cough	356 (98.1)	55 (100.0)	49 (98.0)	252 (97.7)	.523
Fever	218 (60.1)	31 (56.4)	28 (56.0)	159 (61.6)	.631
Night sweats	251 (69.1)	42 (76.4)	39 (78.0)	170 (65.9)	.107
Chills	131 (36.1)	28 (50.9)	14 (28.0)	81 (31.4)	.031
Anorexia	95 (26.2)	18 (32.7)	12 (24.0)	65 (25.2)	.479
Weight loss	275 (75.8)	44 (80.0)	44 (88.0)	187 (72.5)	.047
Previous TB episode	55 (15.2)	8 (14.5)	10 (20.0)	37 (14.3)	.588
Rifampicin resistant*	58 (19.1)	8 (19.0)	7 (17.1)	43 (19.5)	.934
Mycobacterial load†					.017
Very low	47 (13.4)	5 (9.4)	4 (8.2)	38 (15.3)	
Low	81 (23.1)	6 (11.3)	12 (24.5)	63 (25.3)	
Medium	121 (34.5)	16 (30.2)	18 (36.7)	87 (34.9)	
High	102 (29.1)	26 (49.1)	15 (30.6)	61 (24.5)	
Weight, kg, mean (SD)	50.6 (8.6)	50.1 (6.9)	49.3 (9.1)	51.0 (8.8)	.078
BMI, kg/m^2^, mean (SD)	18.9 (3.3)	17.9 (2.4)	18.2 (4.3)	19.2 (3.2)	<.001
Temperature, °C (n = 361), mean (SD)	35.9 (1.4)	36.1 (1.3)	35.7 (2.2)	35.9 (1.2)	<.001

BMI = body mass index, TB = tuberculosis, SD = standard deviation.

* Only 303 had a drug susceptibility test.

† Only 351 participants had grading of the mycobacterial load.

### 3.2. Comparison of monocyte, neutrophil, CD4+ and CD8+ T-lymphocyte counts, and CD4:CD8 ratio with and without smoking history

The median (interquartile range [IQR]) monocyte count among current smokers was 815 (540–1425) cells/mm^3^ and was significantly higher than that among past smokers (610 (350–900) cells/mm^3^, *P* = .017) and never smokers (560 [400–800] cells/mm^3^, *P* = .001). Additionally, current smokers had significantly higher median (IQR) neutrophil (5570 [4030–8405] vs 4250 [2730–6460] cells/mm^3^, *P* = .017) and CD4+ T-cell counts (512 [307–770] vs 367 [172–613] cells/mm^3^, *P* = .008) than never smokers. Other pairwise intergroup comparisons were not statistically significant. Table 2 shows the cell counts and CD4:CD8 ratio compared across the three categories of smoking status.

**Table 2:** Comparison of monocyte, neutrophil, CD4 and CD8 T-cell counts, and CD4:CD8 ratio by smoking status.

Parameter	Current smokers (n = 55)	Past smokers (n = 50)	Never smokers (n = 258)	*P* value
Monocytes*, median (IQR), (n = 266)	815 (540–1425)	610 (350–900)	560 (400–800)	.011
Neutrophils*, median (IQR), (n = 266)	5570 (4030–8405)	4100 (2750–5500)	4250 (2730–6460)	.028
CD4+ T-cells*, median (IQR)	512 (309–770)	367 (172–613)	446 (227–741)	.077
CD8+ T-cells*, median (IQR)	406 (239–647)	426 (226–656)	423.5 (269–607)	.761
CD4: CD8, median (IQR)	1.21 (0.76–2.08)	0.97 (0.35–1.57)	1.24 (0.39–1.91)	.472

* Cells/mm^3^.

IQR = interquartile range.

### 3.3. Correlation between monocyte, neutrophil, CD4 and CD8+ T-lymphocyte counts, and CD4:CD8 ratio with number of cigarettes smoked

The median (IQR) number of cigarettes smoked among current smokers were 5 (3–10) per day. Among past smokers, the median (IQR) number of cigarettes smoked were 5.5 (3–13) per day. The monocyte counts positively correlated with the number of cigarettes smoked per day among current smokers (*R* = 0.43, *P* = .006). The correlation between cigarettes smoked and other cell counts and CD4:CD8 ratio were not statistically significant (Table 3).

**Table 3:** Correlation between the monocyte, neutrophil, CD4 and CD8 T-cell counts, and CD4:CD8 ratio with the number of cigarettes smoked per day.

	Current smokers		Past smokers	
	Correlation coefficient (*r*)	*P* value	*r*	*P* value
Monocyte count	0.43	0.006*	0.14	.490
Neutrophil count	0.17	0.309	0.06	.784
CD4+ T-cells	0.07	0.623	–0.17	.303
CD8+ T-cells	0.08	0.593	0.00	.987
CD4:CD8	0.00	0.987	–0.11	.513

* Statistically significant result.

### 3.4. Factors associated with current smoking among people with TB

In a multivariable logistic regression model that adjusted for HIV, CD4+, CD8+ T-cell and neutrophil counts, and the CD4:CD8 ratio, an increase in the monocyte count was associated with current cigarette smoking (adjusted odds ratio [aOR] = 4.82, 95% confidence interval [CI] 1.61–14.39, *P* = .005). Other factors associated with higher odds of current smoking (Table 4) were any history of alcohol use (aOR = 3.07, 95% CI 1.27–7.39, *P* = .012) and male sex (aOR = 11.70, 95% CI 3.07–44.54, *P* < .001).

**Table 4:** Factors associated with current cigarette smoking among TB patients.

Variable	Crude odds ratio (95% CI)	*P* value	Adjusted odds ratio (95% CI)	*P* value
Sex				
Male	10.08 (3.56, 28.59)	<0.001	11.70 (3.07, 44.54)	<.001
Female	ref		ref	
Alcohol use				
Ever used	4.62 (2.30, 9.27)	<0.001	3.07 (1.27, 7.39)	.012
Never used	ref		ref	
HIV infection				
Positive	0.51 (0.26, 0.98)	0.044	1.30 (0.42, 4.02)	.643
Negative	ref		ref	
Monocyte count	5.03 (2.48, 10.22)	<0.001	4.82 (1.61, 14.39)	.005
Neutrophil count	1.11 (1.02, 1.21)	0.016	1.01 (0.88, 1.15)	.936
CD4+ T lymphocyte count	1.00 (1.00, 1.00)	0.163	1.00 (1.00, 1.00)	.051
CD8+ T lymphocyte count	1.00 (1.00, 1.00)	0.933	1.00 (1.00, 1.00)	.171
CD4:CD8 ratio	1.20 (0.91, 1.59)	0.194	0.75 (0.34, 1.61)	.456

CI = confidence interval.

### 3.3. Post hoc analysis for factors associated with an increase in the monocyte counts

Current cigarette smoking was independently associated with an increase in the monocyte count (aOR = 1.80, 95% CI 1.39–2.32, *P* < .001). Conversely, being HIV positive (aOR = 0.74, 95% CI 0.60–0.90, *P* = .003) and an increase in the hemoglobin levels (aOR = 0.96, 95% CI 0.90–0.97, *P* < .001) were associated with lower monocyte counts.

## 4. Discussion

The aim of this study was to compare the blood monocyte, neutrophil, CD4+ and CD8+ T-lymphocyte counts, and the CD4:CD8 ratio among three categories of people with pulmonary TB: never smokers, past smokers, and current smokers. We also determined factors associated with current cigarette smoking. Our study adds to the paucity of data on the effect of smoking on peripheral blood cells among people with active TB. We found that current smokers had higher monocyte counts than past or never smokers. Monocyte counts also correlated with the number of cigarettes smoked per day among current smokers. Further, current smokers had higher neutrophil and CD4+ T-lymphocyte counts than never smokers. From our study, the clinical implication of the elevated cell counts is not apparent. Monocytes differentiate into macrophages and dendritic cells to replenish the pool of macrophages in the lungs.^[25]^ Together with CD4+ T-lymphocytes that secrete interferon gamma (IFNɣ), these phagocytes play a central role in TB immune responses.^[25]^ Neutrophils also phagocytose mycobacteria and produce a wide range of cytokines that activate macrophages and CD4+ T lymphocytes against TB.^[26]^ One might expect that higher counts of these immune cells in current smokers would portend an appropriate immune response against *Mycobacterium tuberculosis*. On the contrary, almost half of current smokers had a high sputum mycobacterial load compared to past (30%) and never smokers (25%). Moreover, current smokers had the lowest body mass index (BMI) and highest median temperature than the other groups. Therefore, the elevation in these cells may not be necessarily beneficial in protection against TB. Smoking is known to reduce phagocytic activity of phagocytes and production of IFNɣ from CD4+ T-lymphocytes in TB patients.^[27]^ Thus, the elevation in monocytes, neutrophils and CD4+ T-lymphocytes in TB patients who are current smokers supports the understanding that smoking causes systemic inflammation as observed in the general population of smokers.^[14,28]^

At multivariable analyses, smoking was associated with an increase in blood monocyte but not with neutrophils, CD4+ and CD8+ T-lymphocytes and the CD4:CD8 ratio. Interestingly, there was a dose- and time-dependent relationship between smoking exposure and the monocyte count. That is, the monocyte count increased with the number of cigarettes smoked and current smokers had the highest counts while never smokers had the lowest. The difference in the monocyte count between current and former smokers suggest that smoking cessation results in a progressive reduction of the monocytes as has been observed in cohorts of smokers.^[15,29]^ In a Danish cohort of smokers, the decline in the monocyte counts was very modest in the first one year after smoking cessation while neutrophils and lymphocytes had a more dramatic decline.^[15]^ This could also explain why the differences in the cell counts was only consistent across monocytes given the short cut off period (6 months) used in differentiating the groups. This, however, needs to be confirmed in a cohort of TB patients with history of smoking. Evaluating the trend of the monocyte count in TB patients who smoke is particularly important because of the potential association of high monocyte counts with cardiovascular disease.^[30]^ Moreover, monocytes and neutrophils synergistically interact to accelerate atherosclerosis by neutrophil-driven recruitment of monocytes into sites of lipid accumulation.^[31]^ Since people with TB are at a 51% higher risk of major cardiovascular events than normal controls, future studies could evaluate whether monocytosis has a predictive role among TB patients who smoke.^[32]^ This may also be important among TB survivors since cardiovascular disease accounts for at least 20% of post TB mortality.^[33]^

We found that male sex and alcohol use were also associated with smoking. Male TB patients report higher smoking rates than females and high rates of concurrent smoking and alcohol use is reported among TB patients elsewhere.^[21,34]^ We also observed lower levels of education and BMI among smokers as has been reported by Wessels et al in South Africa.^[34]^ Smoking cessation programs such as nicotine replacement and psychotherapies among people with TB may need to target men and be integrated with alcohol quitting programs.^[35,36]^ However, the efficacy and cost-effectiveness of such programs needs to be evaluated in randomized controlled trials.

A key limitation to our study is the use of self-reports in assessing smoking status. This can cause misclassification, recall, and social desirability biases. However, smoking self-reports among people with TB correlate well with plasma levels of nicotine with a sensitivity of 89%.^[21]^ We therefore believe that this did not significantly affect our categorization of patients. Another limitation is the small population of current and past smokers in comparison to nonsmokers. This could have limited our ability to detect subtle differences in these sub-groups. Nonetheless, the strength of the study was that we were able to demonstrate a dose and time-dependent relationship between the monocyte counts and smoking, supporting the understanding that while cigarettes increase the monocyte counts, smoking cessation reduces the cell counts.

## 5. Conclusion

Cigarette smoking is associated with an increase in the blood monocytes in people with TB in a dose and time-dependent manner. Current smokers also have higher CD4+ T-lymphocytes and neutrophils than never smokers. Notwithstanding, current smokers had high sputum mycobacterial load, the lowest BMI, and highest median temperature. While the clinical significance of the elevated cell counts is not apparent from our data, the findings suggest that current smokers have systemic inflammation that is not necessarily beneficial to TB control in TB patients.

## Author contributions

**Conceptualization:** Joseph Baruch Baluku.

**Data curation:** Joseph Baruch Baluku.

**Formal analysis:** Joseph Baruch Baluku, Martin Nabwana.

**Investigation:** Grace Kansiime, Edwin Nuwagira.

**Methodology:** Joseph Baruch Baluku, Martin Nabwana, Grace Kansiime, Edwin Nuwagira.

**Resources:** Joseph Baruch Baluku.

**Writing – original draft:** Joseph Baruch Baluku.

**Writing – review & editing:** Joseph Baruch Baluku, Grace Kansiime, Edwin Nuwagira.
